# Systematic Analysis Identifies a Specific RNA-Binding Protein-Related Gene Model for Prognostication and Risk-Adjustment in HBV-Related Hepatocellular Carcinoma

**DOI:** 10.3389/fgene.2021.707305

**Published:** 2021-08-04

**Authors:** Maoshi Li, Zhongwei Liu, Jing Wang, Huimin Liu, Hongmei Gong, Shilian Li, Ming Jia, Qing Mao

**Affiliations:** ^1^Department of Infectious Diseases, Southwest Hospital, Army Medical University, Chongqing, China; ^2^Chongqing Key Laboratory for Research of Infectious Diseases, Chongqing, China

**Keywords:** hepatocellular carcinoma, HBV, RNA-binding protein, prognosis, signature, recurrence

## Abstract

**Objective:**

Increasing evidence shows that dysregulated RNA binding proteins (RBPs) modulate the progression of several malignancies. Nevertheless, their clinical implications of RBPs in HBV-related hepatocellular carcinoma (HCC) remain largely undefined. Here, this study systematically analyzed the associations of RBPs with HBV-related HCC prognosis.

**Methods:**

Based on differentially expressed RBPs between HBV-related HCC and control specimens, prognosis-related RBPs were screened by univariate analyses. A LASSO model was then created. Kaplan-Meier curves, ROCs, multivariate analyses, subgroup analyses and external verification were separately applied to assess the efficacy of this model in predicting prognosis and recurrence of patients. A nomogram was created by incorporating the model and clinical indicators, which was verified by ROCs, calibration curves and decision curve analyses. By CIBERSORT algorithm, the association between the risk score and immune cell infiltrations was evaluated.

**Results:**

Totally, 54 RBPs were distinctly correlated to prognosis of HBV-related HCC. An 11-RBP model was created, containing POLR2L, MRPS12, DYNLL1, ZFP36, PPIH, RARS, SRP14, DDX41, EIF2B4, and NOL12. This risk score sensitively and accurately predicted one-, three- and five-year overall survival, disease-free survival, and progression-free interval. Compared to other clinical parameters, this risk score had the best predictive efficacy. Also, the clinical generalizability of the model was externally verified in the GSE14520 dataset. The nomogram may predict patients’ survival probabilities. Also, the risk score was related to the components in the immune microenvironment.

**Conclusion:**

Collectively, RBPs may act as critical elements in the malignant progression of HBV-related HCC and possess potential implications on prognostication and therapy decision.

## Introduction

Hepatocellular carcinoma (HCC) is the most prevalent type of liver cancer and represents a common malignant neoplasm globally (Liu et al., 2019). Because of the high risk of recurrence and metastasis, the 5-year survival probabilities of advanced HCC are still undesirable. The epidemiology of HCC is affected by underlying liver diseases especially hepatitis B virus (HBV) (Chabrolles et al., 2020). It has been estimated that HBV infection is responsible for 50% of HCC worldwide (Li et al., 2019). HBV that integrates into cancer-relevant genes may drive hepatocarcinogenesis (Nakagawa et al., 2019). Nevertheless, the mechanism by which HBV infection contributes to HCC remain deficiently expounded (Torresi et al., 2019). The extensive decrease in HCC cases demands a broader range of universal HBV vaccination application and efficient therapy of HBV-relevant chronic hepatitis, which has a long way to go (Sagnelli et al., 2020).

RNA-binding protein (RBP) represents a critical mediator of cancer phenotype (Müller-McNicoll and Neugebauer, 2013). RBP acts dynamically and multifunctionally on multiple levels of post-transcriptional gene expression, such as mRNA splicing, stability and translation (Hou et al., 2019). Over 1,500 human RBPs have been discovered, which possess 600 structure-specific RNA-binding domains (Schneider et al., 2019). Most of them are characterized by evolutionary conservatism and ubiquitous expression to maintain cellular homeostasis (Chabrolles et al., 2020). Genetic and proteomic data highlight that alterations in RBP expression display profound implications on HCC (Lin et al., 2019). For instance, RBP YTHDF2 facilitates cancer stem cell phenotype and metastasis in HCC through regulating OCT4 N^6^-methyladenosine methylation (Zhang et al., 2020). RBP RPS3 leads to hepatocarcinogenesis through up-regulating SIRT1 at a post-transcriptional level (Zhao et al., 2019). RBP RBM3 induces proliferation of HCC cells *via* regulating circRNA-SCD production (Dong et al., 2019). Despite this, the functions of most RBPs in HBV-related HCC remain still unclear.

This study systematically dissected the prognosis-related RBPs of HBV-related HCC, and established and externally verified an RBP model for predicting prognosis and recurrence by applying least absolute shrinkage selection operator (LASSO). This model might serve as a potential prognostic stratification tool and offer several therapeutic targets for HBV-related HCC.

## Materials and Methods

### Data Acquisition

RNA-seq transcriptome data and complete clinical information of 374 HCC and 50 control specimens were retrieved from the Cancer Genome Atlas (TCGA) database^1^. Among them, 108 HBV-related HCC were extracted from TCGA dataset, which were employed as the discovery set. Expression profiling and clinical features of 224 HBV-related HCC samples were obtained from the GSE14520 dataset in the Gene Expression Omnibus (GEO;^2^) repository. This GSE14520 dataset was based on the GPL571 and GPL3921 platforms. This dataset was used as the validation set. Table 1 listed the clinical characteristics of HBV-related HCC patients. Each probe was transformed to the corresponding gene symbol. If multiple probes matched the same gene symbol, the average value was determined as the expression value of this gene. Based on published articles, a list of 1,542 RBPs was collected (Supplementary Table 1).

**TABLE 1:** Clinical characteristics of HBV-related HCC patients.

Characteristics	TCGA (*n* = 108)	GSE14520 (*n* = 224)
**Age**		
<65	81	199
≥65	27	25
**Gender**		
Male	89	195
Female	19	29
**Survival status**		
Alive	20	86
Dead	88	138
**TNM stage**		
Stage I	72	96
Stage II	21	78
Stage III	11	50
Stage IV	2	0
**Recurred status**		
Yes	45	125
No	56	99

### Differential Expression Analysis

Differences in expression levels of RBPs were analyzed between 108 HBV-related HCC and 50 control samples *via* limma package based on RNA-seq transcriptome data (Ritchie et al., 2015). Under the threshold of | fold-change| > 2 and adjusted *p* < 0.001, up- and down-regulated RBPs were screened for HBV-related HCC.

### Functional Annotation Analysis

Gene ontology (GO) enrichment analysis primarily contains three categories: biological process (BP), cellular component (CC) and molecular function (MF). Furthermore, Kyoto Encyclopedia of Genes and Genomes (KEGG) can provide signaling pathways involved in RBPs. Here, GO, and KEGG enrichment analyses of differentially expressed RBPs were carried out *via* clusterProfiler package (Ashburner et al., 2000). Terms with false discovery rate (FDR) < 0.05 were significantly enriched by above RBPs. Functional association between HBV-related RBPs was analyzed through the STRING database^3^ (Szklarczyk et al., 2017). A protein-protein interaction (PPI) network was conducted utilizing Cytoscape software (Doncheva et al., 2019).

### Determining Candidate Prognosis-Related RBPs

Univariate Cox regression analysis was applied for analyzing the associations between overall survival (OS) of HBV-related HCC patients and differentially expressed RBPs utilizing survival package. Prognosis-related RBPs were determined with the threshold of *p* < 0.05. Then, key prognosis-related RBPs were screened through LASSO regression analysis with glmnet package (Engebretsen and Bohlin, 2019). Normalized regression coefficients of key prognosis-related RBPs were calculated based on multivariate regression analysis.

### Establishment of an RBP-Related Prognostic Model

A risk score was established on the basis of the key prognosis-related RBPs for HBV-related HCC patients in the TCGA dataset. This study calculated the risk score of each patient, according to the formula: risk score = expression of RBP1 × regression coefficient of RBP1 + expression of RBP2 × regression coefficient of RBP2 + … + expression of RBPn × regression coefficient of RBPn. According to the median risk score, HBV-related HCC patients were assigned into high- and low-risk groups. Hierarchical clustering analysis was applied for showing the associations between expression patterns of above RBPs and clinical characteristics (stage, grade, gender, and age). Kaplan-Meier curve analyses were presented for investigating the differences in OS, disease-free survival (DFS) and progression-free interval (PFI) between two groups using Survival package. P values were determined with log-rank tests. Time-dependent receiver operating characteristic (ROC) curves under one-, three- and five-year OS, DFS, and PFI were generated by SurvivalROC package (Heagerty et al., 2000). Then, the area under the curve (AUC) was calculated to evaluate the predictive usefulness of the risk score.

### Prognostic Model Verification

The associations between risk score, age, gender, grade, stage, and prognosis were evaluated utilizing univariate cox regression analysis. Prognostic factors with *p* < 0.05 were incorporated for multivariate cox regression analysis. Independent prognostic factors were then identified for HBV-related HCC. Time-independent ROCs of risk score, age, gender, grade, and stage were separately constructed and the predictive power was compared. In published literature, Fang and Chen (2020) proposed a two-m^6^A RNA methylation regulator prognostic model (HNRNPA2B1 and RBM15) for HBV-related HCC prognosis. By ROCs, the predictive efficacy was compared with our prognostic model. For evaluating the clinical generalizability of the model, this model was verified in the GSE14520 dataset. With the same formula, the risk scores of patients were calculated in this dataset. Based on the median risk score, OS differences between high- and low-risk groups were assessed by Kaplan-Meier curves and log-rank tests. The predictive performance was verified by ROCs.

### Subgroup Analysis

HBV-related HCC subjects in TCGA dataset were stratified into subgroups according to clinical characteristics, as follows: age ≥ 65 and < 65 subgroups, female and male subgroups, grade 1–2 and 3–4 subgroups and stage I-II and stage III-IV subgroups. In each subgroup, Kaplan-Meier curves of OS were conducted between high- and low-risk patients. Survival differences were estimated with log-rank tests.

### Nomogram Construction

A nomogram by incorporating gender, age, grade, stage, and risk score was created for predicting one-, three-, and five-year survival probabilities of HBV-related HCC patients in the TCGA dataset. The accuracy in predicting prognosis was evaluated by ROC curves, calibration curves and decision curve analysis (DCA) (Vickers and Elkin, 2006). ROC curves were conducted for evaluating the predictive performance of this nomogram for one-, three- and five-year OS. By calibration curves, discrepancy between nomogram-estimated and actual one-, three-, and five-year survival duration was analyzed. DCA was applied for quantifying the clinical practical use with survival outcomes of a decision considered.

### Gene Set Enrichment Analyses (GSEA)

HBV-related HCC specimens were stratified into high- and low-risk groups. By Gene Set Enrichment Analyses (GSEA)^4^, potential mechanisms of this prognosis-related model were elucidated (Subramanian et al., 2005). GSEA was carried out for finding enriched KEGG pathways, with KEGG gene set “c2.cp.kegg.v7.0.symbols.gmt” as a reference. Pathways with nominal *p* < 0.05 were distinctly enriched.

### Estimation of Immune Cell Infiltrations and HLA Expression

By applying CIBERSORT algorithm^5^, the proportions of 22 immune cells in HBV-related HCC and control specimens were quantified based on their expression profiling (Newman et al., 2015). The proportions of all immune cells in each specimen were equal to 1. The LM22 signature was employed as a reference set. The permutations were set as 1,000. Samples with *p* < 0.05 were screened for further analyses. The correlations between risk score and infiltrations of immune cells and HLA family expression were assessed with Spearson correlation analysis.

## Results

### Expression and Functions of RBPs in HBV-Related HCC

**FIGURE 1:**
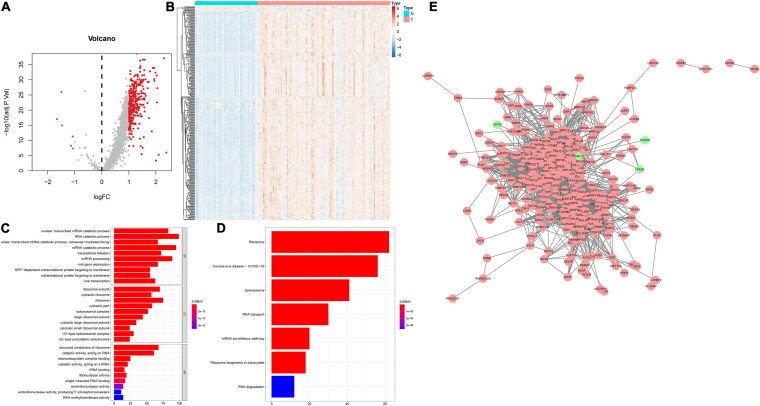
Expression patterns and functions of RBPs in HBV-related HCC tissues. **(A)** Volcano plots of up- and down-regulated RBPs in HBV-related HCC than control specimens. Red dots represented up-regulated RBPs; blue dots represented down-regulated RBPs; gray dots represented no differentially expressed RBPs. **(B)** Heatmap visualizing expression values of up- (red) and down-regulated (blue) RBPs in HBV-related HCC and control specimens. **(C)** The first ten biological processes (BPs), cellular components (CCs) and molecular functions (MFs) involved in differentially expressed RBPs. **(D)** KEGG pathways enriched by above RBPs. The longer the band, the greater the number of enriched genes. The more the color tended to red, the smaller adjusted p-value. **(E)** A PPI network based on the differentially expressed RBPs. Red indicated up-regulated RBPs and green indicated down-regulated RBPs.

This study collected 1,542 RBPs and analyzed their expression in 108 HBV-related HCC and 50 control tissues. | Fold-change| > 2 and adjusted *p* < 0.001 were set as the screening thresholds. As a result, 340 RBPs were up-regulated in HBV-related HCC than control specimens (Figures 1A,B). The first 20 up-regulated RBPs were shown in Table 2. Meanwhile, there were six down-regulated RBPs (GSPT2, PAIP2B, ANG, AZGP1, CPEB3, and ZFP36) in HBV-related HCC compared to controls (Figures 1A,B and Table 2). These RBPs were primarily enriched in mRNA metabolic biological processes such as nuclear-transcribed mRNA catabolic process, RNA catabolic process, nuclear-transcribed mRNA catabolic process, mRNA catabolic process and ncRNA processing (Figure 1C). Our KEGG analysis demonstrated that above RBPs were distinctly related to key pathways including ribosome, spliceosome, RNA transport, mRNA surveillance pathway, ribosome biogenesis in eukaryotes and RNA degradation, confirming their roles on post-transcriptional gene regulation (Figure 1D). A PPI network was conducted for revealing the interactions between these HCC-related RBPs (Figure 1E).

**TABLE 2:** The first 20 up-regulated RBPs and 6 down-regulated RBPs in HBV-related HCC.

ID	logfold-change	Average expression	t	P	Adjusted p	B
PEG10	2.387979	2.638582	5.173505	6.75E-07	9.80E-07	4.947393
RNASEH2A	2.299935	4.022123	18.10863	1.07E-40	7.58E-38	82.02684
EEF1A2	2.10402	2.672998	4.815884	3.35E-06	4.71E-06	3.396926
TARBP1	2.052569	3.046862	13.85154	3.05E-29	6.00E-28	55.80843
PABPC1L	2.039105	3.485111	12.0097	3.86E-24	2.85E-23	44.13127
EZH2	2.031468	2.209527	15.56872	6.04E-34	3.73E-32	66.57365
RPS4Y1	2.002975	5.389658	3.65882	0.000343	0.000425	−1.01902
DDX39A	1.983421	5.158247	15.98784	4.45E-35	4.21E-33	69.16769
SMG5	1.942159	5.259853	14.82399	6.46E-32	2.41E-30	61.92894
BOP1	1.938054	5.235709	15.10358	1.11E-32	5.44E-31	63.6776
NELFE	1.937597	5.596385	16.63109	8.36E-37	1.19E-34	73.1174
SNRPE	1.883775	6.305086	16.68019	6.18E-37	1.19E-34	73.41722
MSI1	1.847698	1.586866	8.299861	3.97E-14	9.03E-14	21.2786
SNRPB	1.780655	7.311245	16.16782	1.46E-35	1.59E-33	70.27684
RPL22L1	1.747909	4.488432	10.002	1.26E-18	4.14E-18	31.52673
SPATS2	1.733092	2.806658	14.71432	1.29E-31	4.26E-30	61.24148
SF3B4	1.698588	5.667539	14.66409	1.77E-31	5.46E-30	60.92638
RBM3	1.697054	6.272179	13.86538	2.79E-29	5.58E-28	55.89584
PRIM1	1.688578	3.095013	11.94612	5.79E-24	4.17E-23	43.72825
EXO1	1.626191	1.287369	12.87215	1.57E-26	1.87E-25	49.60374
GSPT2	−1.0703	2.210642	−5.84768	2.69E-08	4.25E-08	8.079581
PAIP2B	−1.0761	2.263303	−7.60705	2.20E-12	4.47E-12	17.3157
ANG	−1.13722	9.092962	−6.28555	2.93E-09	4.93E-09	10.24674
AZGP1	−1.469	9.486897	−9.06554	4.04E-16	1.08E-15	25.81354
CPEB3	−1.4956	2.109004	−13.3884	5.81E-28	9.16E-27	52.87762
ZFP36	−1.65925	7.068678	−9.88183	2.67E-18	8.48E-18	30.78536

### Establishing a RBP Model for Predicting HBV-Related HCC Patients’ Prognosis and Recurrence

**FIGURE 2:**
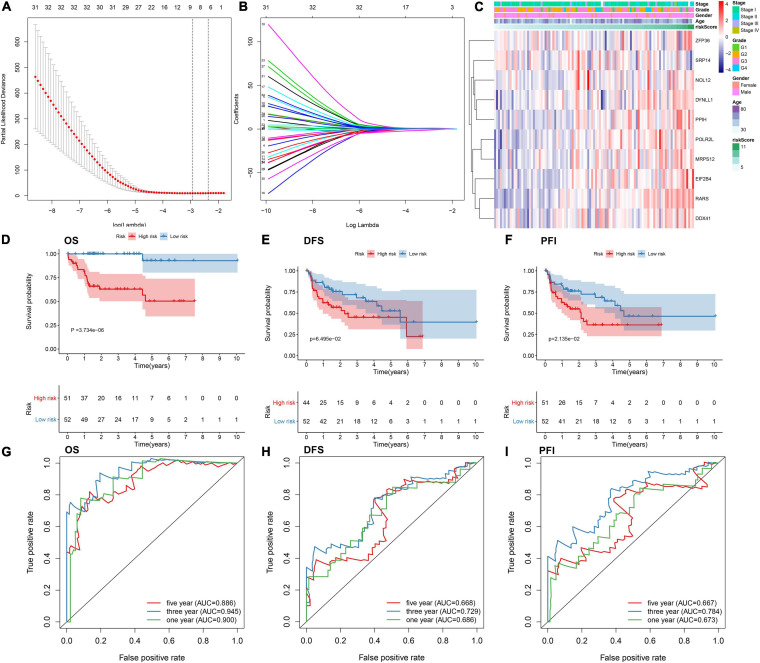
Establishing an RBP prognostic model for HBV-related HCC patients in the TCGA dataset. **(A)** Relationships of λ and partial likelihood deviance. The vertical slash represents the optimal λ value. **(B)** LASSO regression coefficients of RBPs in this model. **(C)** Heatmap for the correlations between expression values of RBPs and clinical features. Kaplan-Meier curves of **(D)** OS, **(E)** DFS and **(F)** PFI between high- and low-risk patients. The ROCs of **(G)** OS, **(H)** DFS, and **(I)** PFI based on the risk score.

By univariate analyses, we investigated which dysregulated RBPs were in relation to HBV-related HCC patients’ survival outcomes. With the cutoff of *p* < 0.05, 54 RBPs were distinctly correlated to prognosis (Table 3). These prognosis-related RBPs were utilized for LASSO analysis. As a result, 10 key RBPs were screened for constructing a prognostic model (Figures 2A,B). The regression coefficients of above RBPs were as follows: POLR2L = 0.453206443450835; MRP S12 = 0.00334358058970182; DYNLL1 = 0.0556454917799665; ZFP36 = 0.215686692231002; PPIH = 0.300574508736105; RARS = 1.0223374037773; SRP14 = −1.08473362012663; DDX 41 = 0.00122492284215762; EIF2B4 = 0.348820298750318; NOL12 = 0.155516694418799. The risk score of each subject from TCGA dataset was determined according to expressions and regression coefficients of RBPs. Heatmap depicted the correlations between expression patterns of POLR2L, MRPS12, DYNLL1, ZFP36, PPIH, RARS, SRP14, DDX41, EIF2B4, and NOL12 and clinical characteristics of HBV-related HCC patients (Figure 2C). Patients from the TCGA dataset were assigned into high- and low-risk groups. As a result, high-risk subjects were indicative of unfavorable OS (*p* = 3.734e-06; Figure 2D), DFS (*p* = 6.495e-02; Figure 2E) and PFI (*p* = 2.135e-02; Figure 2F). The ROCs were plotted for evaluation of the predictive performance. The AUCs under one-, three-, and five-year OS were separately 0.900, 0.945 and 0.886, demonstrating that this model could be precisely predictive of OS probabilities (Figure 2G). Meanwhile, the AUCs under one-, three-, and five-year DFS were 0.686, 0.729 and 0.668 (Figure 2H) as well as the AUCs under one-, three-, and five-year PFI (Figure 2I) were 0.673, 0.784, and 0.667, which were indicative that this model possessed the well performance on predicting HBV-related HCC recurrence and progression.

**TABLE 3:** Prognosis-related RBPs in HBV-related HCC patients by univariate cox regression analysis.

ID	HR	HR.95L	HR.95H	P	ID	HR	HR.95L	HR.95H	P
DDX41	4.4441	1.4451	13.667	0.0093	UTP6	2.6888	1.1424	6.3284	0.0235
NSUN5	2.6559	1.0087	6.9926	0.048	GPATCH4	2.1814	1.1159	4.2646	0.0226
ILF2	1.9392	1.0166	3.6988	0.0444	MRPS12	2.5059	1.3783	4.5562	0.0026
SNRPA	2.4521	1.1227	5.3556	0.0244	RNF113A	2.0476	1.052	3.9855	0.0349
SRP14	0.3565	0.132	0.963	0.0419	CNOT10	3.1297	1.5282	6.4095	0.0018
F3	3.027	1.2561	7.2945	0.0136	PHF5A	2.7005	1.1897	6.1301	0.0175
TCOF1	2.7229	1.3076	5.6701	0.0074	FTSJ1	2.3828	1.1602	4.894	0.0181
METTL5	2.3697	1.0282	5.4613	0.0428	NOP2	2.4581	1.0643	5.677	0.0352
DYNLL1	3.4144	1.3015	8.9577	0.0126	PPIH	2.6265	1.4203	4.8573	0.0021
NHP2	3.0865	1.3129	7.2564	0.0098	NPM1	2.1356	1.0892	4.1871	0.0272
RRP36	2.3214	1.0739	5.0183	0.0323	METTL1	1.9349	1.0773	3.4753	0.0272
PA2G4	2.8337	1.1739	6.8404	0.0205	PES1	2.1902	1.0737	4.4676	0.0311
RUVBL2	3.0262	1.3085	6.9991	0.0096	RNASEH1	3.0569	1.2504	7.4733	0.0143
POP4	2.1925	1.139	4.2203	0.0188	BUD13	2.2305	1.0776	4.6167	0.0307
EIF3B	2.4609	1.2188	4.9692	0.012	TAF9	2.3771	1.2161	4.6465	0.0113
THOC3	2.8281	1.337	5.9822	0.0065	HNRNPAB	2.8933	1.1947	7.0072	0.0186
HARS2	4.8941	2.1092	11.356	0.0002	RPP40	2.0237	1.0355	3.9551	0.0392
CD2BP2	2.5789	1.0669	6.2337	0.0354	MOV10	3.8285	1.6909	8.6686	0.0013
EIF2B4	4.2515	1.8898	9.5642	0.0005	MRPL33	2.385	1.2963	4.3881	0.0052
LARS	2.3723	1.0776	5.2223	0.0319	TRMT6	2.1637	1.0222	4.5801	0.0437
NOL12	1.9271	1.0658	3.4846	0.03	DKC1	2.1134	1.0423	4.285	0.038
NOP56	2.3817	1.317	4.3069	0.0041	EIF4A3	2.196	1.0119	4.7655	0.0466
WDR4	2.0355	1.0801	3.836	0.0279	POLR2L	2.5862	1.4397	4.6458	0.0015
PTGES3	2.9652	1.1175	7.8681	0.029	BRIX1	2.0967	1.1064	3.9735	0.0232
RARS	8.3614	2.9287	23.872	< 0.0001	ZFP36	1.6328	1.0219	2.6087	0.0403
GEMIN7	1.9608	1.0977	3.5025	0.0229	GAPDH	2.2464	1.2433	4.0587	0.0073
EIF3K	2.0916	1.1255	3.8871	0.0196	RBM24	1.3877	1.0278	1.8737	0.0324

### The RBP Model Displays Independent and Well Predictive Power for HBV-Related HCC Patients

**FIGURE 3:**
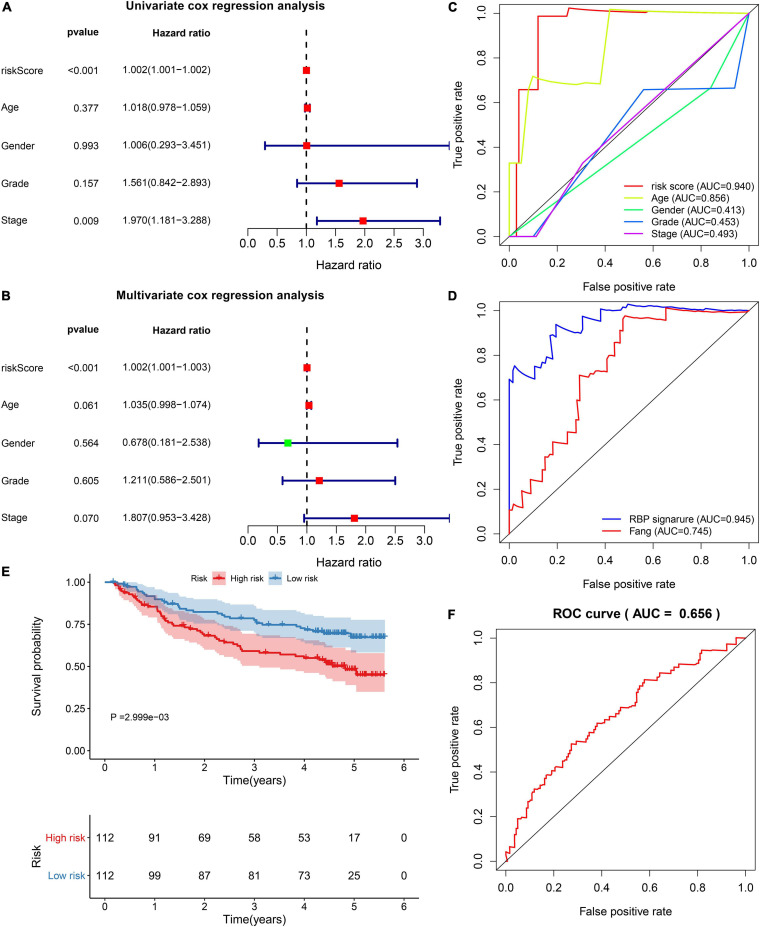
Verification of the independency and accuracy of the prognostic model. **(A)** Univariate and **(B)** multivariate cox regression analyses of risk score, age, gender, grade, and stage with HBV-related HCC patients in the TCGA dataset. **(C)** The ROCs for comparing the AUCs of the risk score with age, gender, grade, and stage. **(D)** The ROCs for comparing the AUCs of the risk score with the model constructed by Fang et al. **(E)** Survival differences between high- and low-risk patients in the GSE14520 dataset. P value was estimated with log-rank test. **(F)** The ROC for evaluation of the predictive performance of the risk score.

By univariate analyses, we investigated the associations between survival outcomes and risk score and clinical parameters in the TCGA dataset. In Figure 3A, risk score (*p* < 0.001) and stage (*p* = 0.009) were both risk factors of HBV-related HCC. Following multivariate analyses, the risk score was independently predictive of survival outcomes (*p* < 0.00l; Figure 3B). In comparison to other clinical parameters, this risk score exhibited the highest AUC of OS (0.940). This demonstrated that the risk score possessed more excellent predictive performance than other parameters (Figure 3C). Compared to the published prognostic model (HNRNPA2B1 and RBM15), higher AUC value was investigated in our model (Figure 3D). We further evaluated the clinical generalizability of this model. In the GSE14520 dataset, high-risk scores were also indicative of unfavorable survival outcomes (*p* = 2.999e-03; Figure 3E) and the AUC value was 0.656 (Figure 3F). Collectively, this model displayed the independent and well power in predicting prognosis.

### Subgroup Analysis of This Prognostic RBP Model in HBV-Related HCC

**FIGURE 4:**
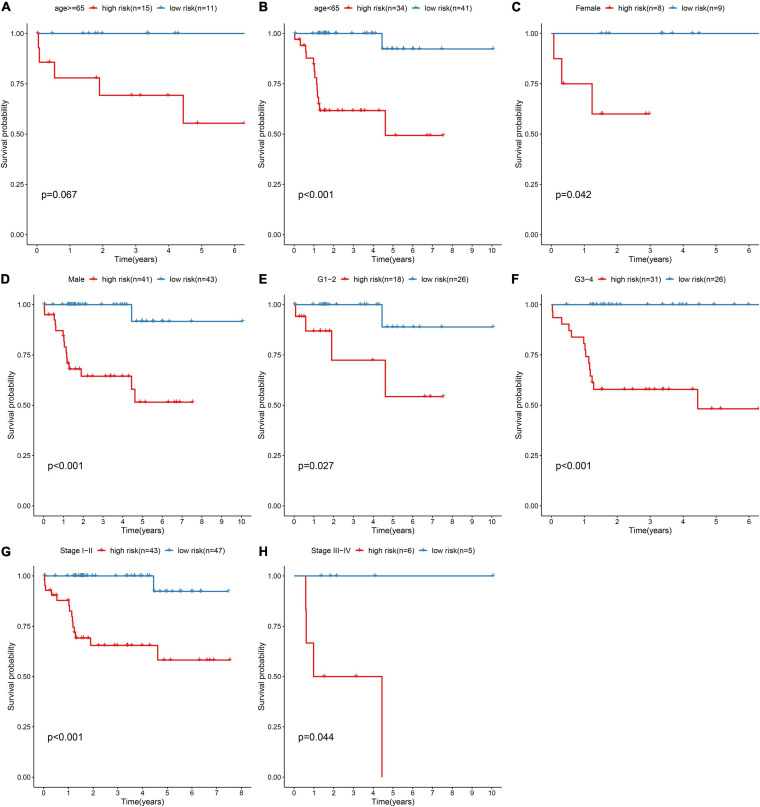
Subgroup analysis for assessing the sensibility of the risk score to predict HBV-related HCC patients’ prognosis. Kaplan-Meier curves for survival outcomes of high- and low-risk patients in **(A)** age ≥ 65 and **(B)** < 65 subgroups, **(C)** female and **(D)** male subgroups, **(E)** grade 1–2 (G1-2) and **(F)** grade 3–4 (G3-4) subgroups, **(G)** stage I–II and **(H)** stage III–IV subgroups. *P* values were estimated with log-rank tests.

For investigating the predictive sensitivity of this model in HBV-related HCC prognosis, survival analyses were carried out in different subgroups. Our data demonstrated that high-risk scores were distinctly predictive of poorer survival outcomes than low-risk score in age ≥ 65 (*p* = 0.067; Figure 4A) and < 65 (*p* < 0.001; Figure 4B) subgroups, female (*p* = 0.042; Figure 4C) and male (*p* < 0.001; Figure 4D) subgroups, grade 1–2 (*p* = 0.027; Figure 4E) and grade 3–4 (*p* < 0.001; Figure 4F) subgroups and stage I-II (*p* < 0.001; Figure 4G) and stage III-IV (*p* = 0.044; Figure 4H) subgroups.

### Constructing a Prognostic Nomogram for HBV-Related HCC

**FIGURE 5:**
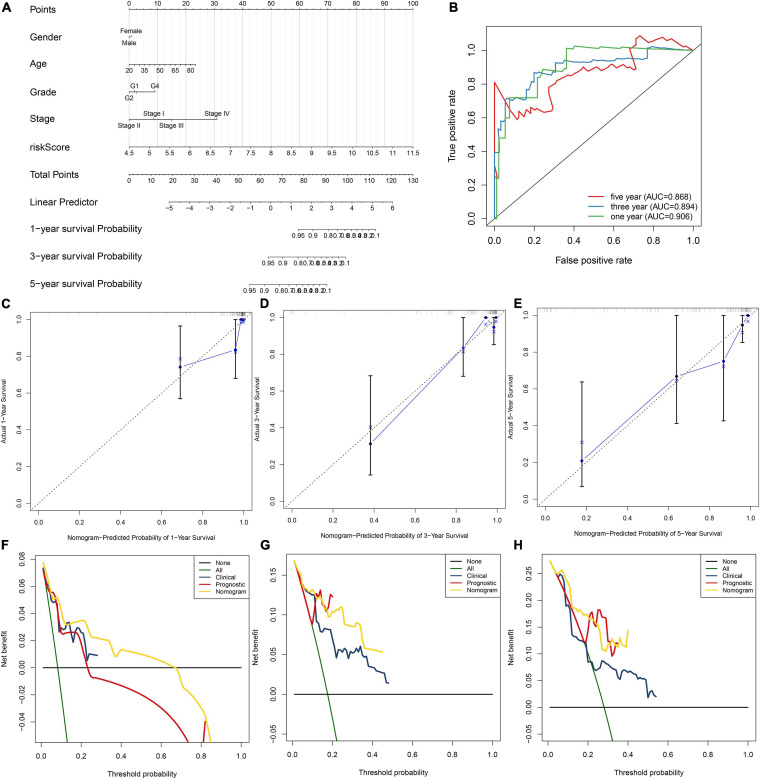
Establishment of the nomogram for HBV-related HCC patients’ prognosis. **(A)** The nomogram that contained gender, age, grade, stage, and risk score for estimating one-, three- and five-year survival probabilities. **(B)** The ROC for evaluation of the predictive performance of this nomogram. **(C–E)** The calibration curves for investigating the discrepancy between nomogram-estimated and actual one-, three- and five-year survival duration. **(F–H)** The DCA for calculating the clinical net benefit of the nomogram, clinical factor, and prognostic factor in comparison to all or none strategies.

To facilitate personalized treatment, we constructed a nomogram by incorporating gender, age, grade, stage, and risk score in TCGA dataset. This nomogram was utilized for estimating one-, three-, and five-year survival probabilities (Figure 5A). ROC curves demonstrated the well performance on predicting one-, three- and five-year (Figure 5B). As shown in our calibration plots, nomogram-estimated one-, three- and five-year survival probabilities were close to actual survival consequences (Figures 5C–E). Meanwhile, DCA showed that this nomogram exhibited the best net benefit for one-, three- and five-year survival duration (Figures 5F–H). Hence, the nomogram model could assist clinical management and decision.

### Activated Pathways in High-Risk HBV-Related HCC

**FIGURE 6:**
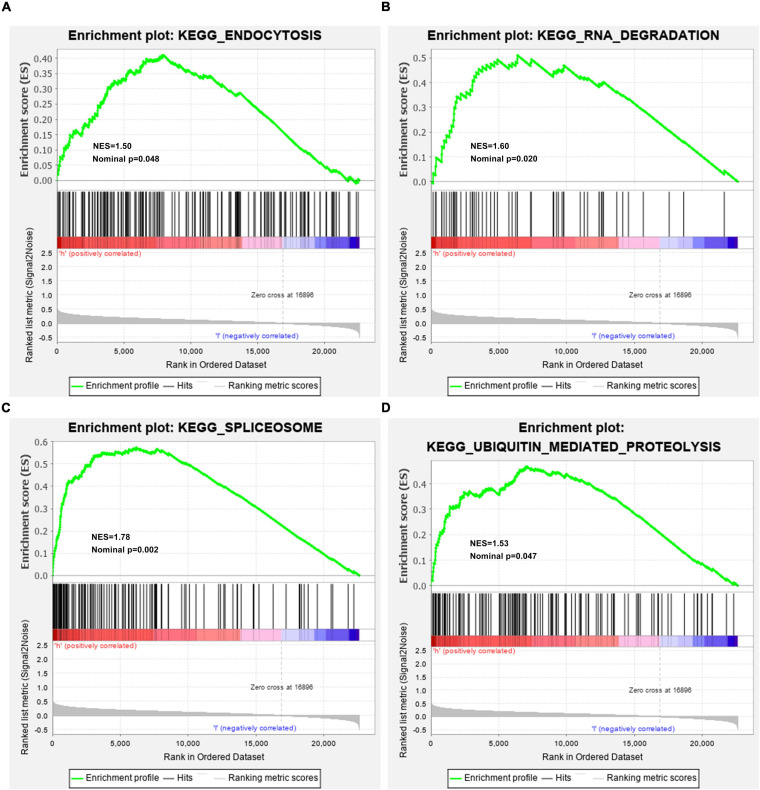
Activated KEGG pathways in high-risk HBV-related HCC samples from the TCGA dataset by GSEA. **(A)** Endocytosis; **(B)** RNA degradation; **(C)** spliceosome; **(D)** ubiquitin-mediated proteolysis.

For observing potential pathways involved in unfavorable survival outcomes, GSEA was carried out. We found that endocytosis (Figure 6A), RNA degradation (Figure 6B), spliceosome (Figure 6C) and ubiquitin-mediated proteolysis (Figure 6D) were distinctly activated in high-risk HBV-related HCC specimens.

### The Risk Score Is Associated With Immune Microenvironment of HBV-Related HCC

**FIGURE 7:**
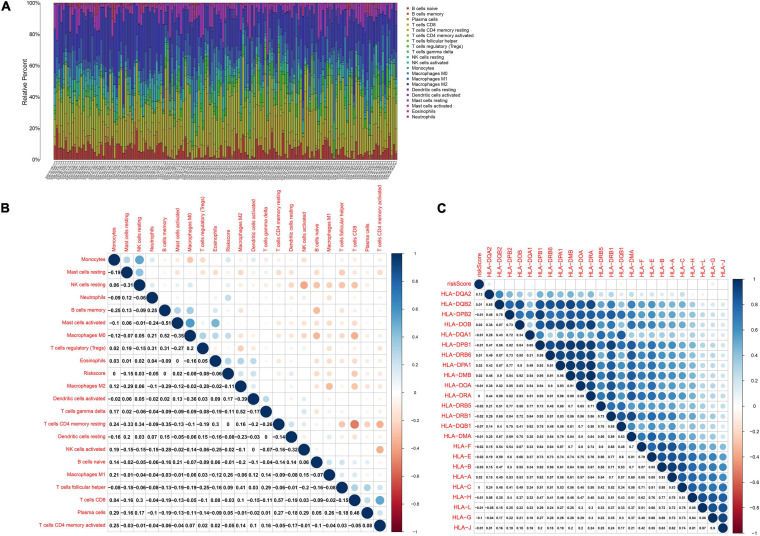
The risk score is related to the immune microenvironment of HBV-related HCC in the TCGA dataset by CIBERSORT. **(A)** The proportions of immune cells in HBV-related HCC samples. **(B)** Heatmap of the correlations between the risk score and immune cells. **(C)** Heatmap visualizing the correlations between the risk score and HLA expression. Correlation coefficients were marked in the box.

CIBERSORT algorithm was applied for inferring the proportions of 22 immune cells in HBV-related HCC tissues, including B cells naïve, B cells memory, plasma cells, T cells CD8, T cells CD4 memory resting, T cells CD4 memory activated, T cells follicular helper, T cells regulatory (Tregs), T cells gamma delta, NK cells resting, NK cells activated, monocytes, macrophages M0, macrophages M1, macrophages M2, dendritic cells resting, dendritic cells activated, mast cells resting, mast cells activated, eosinophils and neutrophils (Figure 7A). There was the heterogeneity in the immune microenvironment among subjects. The close crosstalk between immune cells was found, as shown in Figure 7B. Also, the risk score was associated with mast cells resting, NK cells resting, neutrophils, mast cells activated, macrophages M0, Tregs and eosinophils. Moreover, the risk score displayed the significant correlations to HLA expression in HBV-related HCC specimens (Figure 7C). The data indicated that the risk score was related to the immune microenvironment of HBV-related HCC.

## Discussion

In this study, an RBP-related gene model was created, which could robustly predict prognosis and recurrence of HBV-related HCC individuals. High risk scores were indicative of undesirable survival outcomes. Our data confirmed RBPs as critical elements in the malignant progression of HBV-related HCC. The gene model acted as a key clinical implication in prognostication and therapy decision.

Alterations in RBP expression may lead to carcinogenesis. Here, we identified 340 dysregulated RBPs in HBV-related HCC. These RBPs were distinctly related to mRNA metabolic biological processes such as nuclear-transcribed mRNA catabolic process, RNA catabolic process, nuclear-transcribed mRNA catabolic process, mRNA catabolic process and ncRNA processing as well as key pathways including ribosome, spliceosome, RNA transport, mRNA surveillance pathway, ribosome biogenesis in eukaryotes and RNA degradation. Thus, these RBPs acted as key regulators on post-transcriptional gene expression.

By LASSO analysis, we created an RBP prognostic model, which contained POLR2L, MRPS12, DYNLL1, ZFP36, PPIH, RARS, SRP14, DDX41, EIF2B4, and NOL12. Following external verification, this model possessed higher accuracy and sensitivity on prognostication in comparison to other clinical parameters. The biological implications of above RBPs in this model have been reported in previous research. Liu et al. (2020) found that POLR2L displayed a correlation to survival duration and alternative splicing in lung squamous cell carcinoma patients. MRPS12 functioned as an oncogene and a prognostic candidate in ovarian carcinoma (Qiu et al., 2021). DYNLL1 hypomethylation and upregulation was characterized by stage- and grade-dependent manners and correlated to unfavorable survival outcomes in HCC (Berkel and Cacan, 2020). ZFP36 down-regulation was detected in HCC tissues and served as a tumor suppressor (Kröhler et al., 2019). PPIH was highly expressed in stomach adenocarcinoma and down-regulated PPIH suppressed cellular migratory and invasive behaviors (Li et al., 2021). Also, PPIH up-regulation contributed to undesirable survival outcomes. DDX41 displayed a correlation to tumor stage and grade in HCC (Qi et al., 2020). NOL12 was in relation to kidney renal clear cell carcinoma prognosis (Xiang Y. et al., 2020). Nevertheless, biological roles and clinical implications of these RBPs may require in-depth exploration in HBV-related HCC.

Our results showed that endocytosis, RNA degradation, spliceosome and ubiquitin-mediated proteolysis pathways were markedly activated in high-risk HBV-related HCC specimens, indicating that these pathways might modulate HCC progress and metastasis. RBPs may regulate the stability of mRNAs encoding immune-related proteins, thereby ornamenting the immune microenvironment (Käfer et al., 2019). For instance, RBP ZFP36, as an inflammatory regulator, restrained T cell activation and anti-viral immunity (Moore et al., 2018). RBP YTHDF3 restrained interferon-dependent anti-viral response through increasing FOXO3 translation (Zhang et al., 2019). Suppressing YTHDF1 in dendritic cells induced durable neoantigen-specific immunity and enhanced the efficacy of anti-PD-L1 therapy (Han et al., 2019). PCBP1 served as an intracellular immune checkpoint toward maintaining T cell functions (Ansa-Addo et al., 2020). Recently, the roles of RBPs have been investigated thoroughly in HCC immune microenvironment of HCC. Xiang J et al. (2020) reported that RBP SIRT7 enhanced the efficacies of anti-PD-L1 therapy through MEF2D in HCC cells. Here, our data demonstrated the close associations of the risk score with immune cells in HCC tissues such as mast cells resting, NK cells resting, neutrophils, mast cells activated, macrophages M0, Tregs and eosinophils. More studies will be carried out for verifying the roles of the risk score on ornamenting immune microenvironment in HCC.

## Conclusion

Collectively, this study established an RBP model for predicting OS and recurrence of HBV-related HCC individuals. Following external verification, this model possessed the well predictive efficacy and acted as a robust and specific prognostic indicator. Thus, our findings might assist guide clinical decision and personalized therapies.

## Data Availability Statement

The original contributions presented in the study are included in the article/Supplementary Material, further inquiries can be directed to the corresponding author/s.

## Author Contributions

QM conceived and designed the study. ML, ZL, and JW conducted most of the experiments and data analysis, and wrote the manuscript. HL, HG, SL, and MJ participated in collecting data and helped to draft the manuscript. All authors reviewed and approved the manuscript.

## Conflict of Interest

The authors declare that the research was conducted in the absence of any commercial or financial relationships that could be construed as a potential conflict of interest.

## Publisher’s Note

All claims expressed in this article are solely those of the authors and do not necessarily represent those of their affiliated organizations, or those of the publisher, the editors and the reviewers. Any product that may be evaluated in this article, or claim that may be made by its manufacturer, is not guaranteed or endorsed by the publisher.

## Footnotes

1
https://portal.gdc.cancer.gov/

2
https://www.ncbi.nlm.nih.gov/gds/

3
http://string-db.org/

4
http://software.broadinstitute.org/gsea/index.jsp

5
http://cibersort.stanford.edu/

## Supplementary Material

Supplementary Table 1 A list of 1,542 RBPs.

## Abbreviations

HCC: hepatocellular carcinoma
HBV: hepatitis B virus
RBP: RNA-binding protein
LASSO: least absolute shrinkage selection operator
TCGA: The Cancer Genome Atlas
GEO: Gene Expression Omnibus
BP: biological process
CC: cellular component
MF: molecular function
KEGG: Kyoto Encyclopedia of Genes and Genomes
FDR: false discovery rate
OS: overall survival
DFS: disease-free survival
PFI: progression-free interval
ROC: receiver operating characteristic
AUC: area under the curve
DCA: decision curve analysis
GSEA: Gene Set Enrichment Analyses.
